# High-Intensity Interval Training and Moderate-Intensity Continuous Training Affect Running Economy in Endurance Runners: A Systematic Review and Meta-Analysis of Randomized Controlled Trials

**DOI:** 10.5114/jhk/205427

**Published:** 2025-09-23

**Authors:** Yangya Feng, Daxin Li, Yangli Liu, Donghui Tang

**Affiliations:** 1 College of P.E. and Sport, Beijing Normal University, Beijing, China.; 2 College of P.E. and Sport, Qinghai Normal University, Xining, China.

**Keywords:** athletes, endurance, training methods, aerobic metabolism

## Abstract

This systematic review evaluated the effects of high-intensity interval training (HIIT) and moderate-intensity continuous training (MICT) on running economy (RE) in endurance runners. The search was completed in March 2024 based on research databases, and the language of publication was restricted to English. The primary outcome measure was RE, and it was categorized into three subgroups: Zone 1 (Z1), Zone 2 (Z2), and Zone 3 (Z3). The secondary outcomes assessed were maximal oxygen uptake (VO_2max_) and blood lactate concentration. HIIT significantly improved RE compared to MICT (SMD = 0.44, 95% CI [0.15, 0.72], Z = 3.01, p < 0.05). MICT showed a greater effect on VO_2max_ (MD = 2.48, 95% CI [1.61, 3.34], Z = 5.60, p < 0.05). HIIT was more effective at reducing blood lactate levels (MD = −0.15, 95% CI [−0.28, −0.02], Z = 2.20, p < 0.05). The results indicate that HIIT was more effective than MICT in enhancing RE and delaying lactate accumulation. HIIT can further improve RE and postpone blood lactate accumulation when performed at or below the lactate threshold (≤ Z2). VO_2max_ was more pronounced with MICT. These findings suggest that endurance runners and coaches should choose appropriate methods to optimize physiological adaptations.

## Introduction

In endurance running, the efficiency and economy of energy utilization in human movement are crucial factors that impact performance. This efficiency can be quantified through running economy (RE). RE is defined as the energy expenditure associated with running at submaximal intensities (specific running speeds) and the ratio of work output to oxygen consumption (Saunders et al., 2004; Shaw et al., 2013). It is typically measured by oxygen uptake per unit of body weight over time during exercise (O_2_ ml•kg^-1^•min^-1^) or by oxygen uptake per unit of body weight per distance (O_2_ ml•kg^-1^•km^-1^) (Jones et al., 2021).

Traditional measures of RE have typically been assessed in laboratory settings by having athletes run on a treadmill. In recent years, portable oxygen analyzers have gained widespread use in outdoor settings, providing significant convenience for athletes. RE is influenced by various factors, including the environment, sports biomechanics, physiology, and others (Saunders et al., 2004; Van Hooren et al., 2024). The spring-mass model, in which the spring-like action of the supporting leg counteracts the body’s bounce upon contact with the ground, is a crucial element of RE. Mechanical energy is stored in the muscles, tendons, and ligaments that function across joints. Runners with high running economy (RE) utilize less oxygen at the same steady-state speed compared to runners with low RE (Black et al., 2018). Numerous studies have indicated that trained individuals are more economical than their untrained or less-trained counterparts (Morgan et al., 1995; Unhjem, 2024). It has been demonstrated that training enhances both the morphology and functionality of skeletal muscle mitochondria. At a given submaximal running speed, trained runners utilize less oxygen per mitochondrial respiratory chain due to enhanced respiratory capacity in skeletal muscle. These adaptations lead to a slower rate of muscle glycogen consumption in the active musculature, reduced disruption of homeostasis, and improvements in RE (Holloszy et al., 1977).

In long-distance running, many athletes utilize high-intensity interval training (HIIT) and moderate-intensity continuous training (MICT) to enhance their aerobic capacity and overall athletic performance. HIIT can activate larger motor units, increase the recruitment of fast oxidative and glycolytic muscle fibers (Jastrzębski et al., 2025; Lamboley et al., 2020; Laursen et al., 2007;), induce lactic acid production to stimulate both muscles and the cardiovascular system, enhance muscle pumping function and stroke volume (Dolan et al., 2024; Rakobowchuk et al., 2008; Ramos et al., 2015; Seo et al., 2024), increase mitochondrial volume, and reduce plasma H^+^ concentration (Perry et al., 2008; Ruegsegger et al., 2023). MICT recruits a greater proportion of slow-twitch muscle fibers, enhances mitochondrial content, and increases the oxidative capacity of muscle fibers (Skelly et al., 2021). It also boosts muscle capillary density and improves myocardial perfusion and oxygen delivery (Cocks et al., 2016). HIIT can activate signaling pathways such as peroxisome proliferator-activated receptor gamma coactivator 1-alpha (PGC-1α), a key regulator of mitochondrial biogenesis (Torma et al., 2019), and adenosine monophosphate (AMP) activated kinase (AMPK), an important energy-sensing molecule (Gibala, 2009; Kjøbsted et al., 2018). HIIT promotes mitochondrial biogenesis (Dohlmann et al., 2018) and induces various microRNAs, including miR-486-5p, miR-208b, and miR199a-3p, which significantly enhance muscle protein synthesis and hypertrophy (D’Souza et al., 2018; Grieb et al., 2023). Additionally, HIIT improves fat oxidation and oxygen uptake (Atakan et al., 2022). MICT can activate PGC-1α through calcium/calmodulin-dependent protein kinase II (CaMKII) and the phosphatase calcineurin A (CnA), thereby regulating various downstream targets (Furrer et al., 2023; Laursen, 2010). MICT has a more pronounced effect on the expression of miRNAs, including miR-21, miR-133, miR-19b-3p, and miR-503-5p (Grieb et al., 2023; Widmann et al., 2022). These microRNAs (miRNAs) can regulate myoblast proliferation and differentiation, enhance the oxidative metabolism of slow-twitch muscle fibers, and promote muscle adaptation (Gonçalves et al., 2019; Grieb et al., 2023; Massart et al., 2021; Widmann et al., 2022). Both training modes induce complex changes in microRNAs, with some being regulated exclusively by MICT or HIIT (Grieb et al., 2023; Widmann et al., 2022). HIIT is frequently utilized as an alternative to MICT (Cocks et al., 2016). Consequently, HIIT and MICT have emerged as the most prevalent endurance training strategies (González-Mohíno et al., 2020).

While there is scientific evidence demonstrating that endurance training reduces oxygen uptake, whether interval training is more effective than continuous training remains a debated issue in the literature (Cocks et al., 2016; González-Mohíno et al., 2020). MICT can enhance the body’s capacity to transport and utilize oxygen, improve cardiovascular efficiency, and decrease oxygen uptake during exercise by increasing the adaptability of physiological factors, such as mitochondrial content and capillary density (Cocks et al., 2016; Hesketh et al., 2019). HIIT can induce changes in various physiological factors, including lactic acid accumulation, glycolysis, and oxidative utilization, through varying interval intensities and continuous training protocols (Morais et al., 2025; Sperlich et al., 2010). These changes, in turn, affect mitochondrial function and related proteases, ultimately influencing running economy (González-Mohíno et al., 2020; Schaun et al., 2018). There is no consensus regarding the effects of HIIT and MICT on running economy (González-Mohíno et al., 2020; Wang and Wang, 2024). Several studies have investigated the effects of HIIT and MICT on improving running economy (Schaun et al., 2018; Skovgaard et al., 2018a). Some studies employing similar training strategies found no significant improvements (Parmar et al., 2021; Wang and Wang, 2024). Other researchers have noted that improvements in running economy occur only with low-intensity training (González-Mohíno et al., 2020; Zinner et al., 2018). It remains uncertain whether HIIT has a comparable impact on running economy. This study investigated the effects of HIIT and MICT, summarizing the changes in physiological indices such as maximal oxygen uptake, running economy, and lactate threshold to provide insights into scientific training and heretical basis for endurance runners.

## Methods

### Search Strategy

The processes outlined here were based on the PRISMA Statement (Page et al., 2021), and the registration number is INPLASY2024110120. We searched electronic databases, including PubMed, Embase, Scopus, and Web of Science, from their inception to March 2024 using the following search phrases along with Boolean operators: (running OR jogging OR “marathon running” OR “distance running” OR “endurance running” OR “distance runners” OR “endurance runners” OR “middle distance runners”) AND (“running economy” OR “energy metabolism” OR “metabolism energy” OR “energy expenditure” OR “energy expenditures” OR “expenditure energy” OR energy OR “energy cost” OR “metabolic cost”) AND (“intermittent training” OR “interval exercise” OR “interval running” OR “sprint interval training” OR “intensity training”). The language of publication was restricted to English, and the publication type was restricted to randomized controlled trials (RCTs).

### Inclusion and Study Selection

Inclusion criteria were developed according to PICOS principles. Studies that satisfied the following criteria: (1) participants were endurance runners (P), (2) the intervention was interval training (I) compared with continuous training (C) in the control group, (3) studies reported outcomes related to running economy or energy consumption (O), (4) RCTs published in journals with peer review (S), (5) the study utilized a run-based test course or a run-based training program; were considered in the present review and meta-analysis.

Studies were excluded if they (1) lacked a specified intervention type or were not randomized controlled trials, (2) did not adhere to the established guidelines for a training design, (3) involved training interventions lasting less than four weeks (this criterion was implemented because neuromuscular adaptations have been observed in trained individuals within as little as four weeks, the surface electromyography activity of muscles and the hypertrophy of muscles increased significantly at the 4^th^ week) (Baroni et al., 2013; Del Vecchio et al., 2019; Mayhew et al., 1995; Moritani and deVries, 1979), or if they involved non-interval training interventions. Furthermore, this systematic review did not include any review articles.

### Outcome Measurements

The test results included running economy (RE) as the primary outcome, which was calculated using energy cost or steady-state oxygen consumption. Secondary outcome measures included at least one of the following: VO_2max_ and/or blood lactic acid levels.

### Data Collection Process

The included studies were screened by the Endnote X9 citation manager (Liu et al., 2024). Two researchers independently reviewed this literature, excluded duplicate and irrelevant studies, and then independently decided whether to include them in the study. In the event of disagreement, a third researcher was consulted to make the final decision. First, the titles and abstracts were reviewed to exclude studies that clearly did not meet the inclusion criteria. Then, the full texts of the remaining manuscripts were screened and read, the eligibility was evaluated, and the final literature to be included was determined. Two researchers independently extracted studies that met the inclusion and exclusion criteria, including article publication information, participants’ characteristics, the study design, the research content, intervention-related information, and RE results. The authors of the considered papers were contacted for clarification of incomplete or missing data.

We used Excel to store extracted data. Detailed data were registered, including the first author, the publication year, the country of publication, subjects’ characteristics, VO_2max_, training interventions, methods, running economy, and other relevant factors. We utilized the post-intervention mean and standard deviation comparison between groups for calculating effect sizes. When no standard deviations (SDs) were available, they were calculated from standard errors (SEs), CIs, t or *p* values.

### Risk of Bias Assessment

Two reviewers independently assessed the risk of bias, and any disagreements were resolved through discussion with a third author. The bias in the included randomized controlled trials was evaluated using the Cochrane Risk of Bias Tool, which categorized each item as of low risk, high risk, or unclear risk of bias concerning random sequence generation, allocation concealment, blinding of participants and personnel, blinding of outcome assessment, incomplete outcome data, selective reporting, and other biases (Higgins and Green, 2011).

The performance bias item was eliminated because it was not practicable to blind the participants. A technique was established to assess the potential for bias in selection (random sequence generation and allocation concealment), detection, attrition, and reporting biases. The study was classified as of “high risk” or “low risk”, and if the information was omitted, it was graded as “unclear”. We addressed the impact of bias levels on the weights in our analyses by conducting sensitivity analyses that excluded studies with high risk of bias, and we found that the overall results remained robust. When a significant result (*p* < 0.05) was observed, a funnel plot test was also performed to assess publication bias. The effects of both HIIT and MICT on running economy performance were studied through the funnel plot.

### Statistical Analyses

We conducted a meta-analysis using RevMan 5.3.5 to determine whether HIIT affected running economy performance. For continuous outcomes, we utilized the sample size, mean, and standard deviation (SD) values from both the experimental and control groups. We calculated Hedges’ g, which represented standardized mean differences (SMD), to assess the magnitude of changes in outcomes between the pre-training and post-training periods (Nakagawa and Cuthill, 2007). Additionally, we employed inverse variance weighting to assign weights to each study included in the meta-analysis, with studies contributing more to the pooled effect size when they were of higher quality (e.g., larger sample size, lower risk of bias). To minimize the influence of studies with high risk of bias, lower weights were assigned to studies with methodological limitations or small sample sizes. A random effects model was utilized in the forest plot to synthesize the effect sizes for each group. We aggregated these effect sizes according to the criteria outlined in the Cochrane Handbook when the included publications assessed running economy at various velocities or included multiple training groups (Higgins et al., 2023). Then, statistical heterogeneity was discussed using the I^2^ statistic, where values of 25%, 50%, and 75% indicated low, moderate, and high heterogeneity, respectively (Higgins et al., 2003). The synthesis of the included trials was considered to exhibit significant heterogeneity if *p* < 0.1 or I^2^ > 0.5, prompting the use of a random effects model. To further assess the robustness of the findings and ensure that potential bias did not significantly affect the pooled results, sensitivity analyses were conducted by systematically excluding each trial one by one. The results were visualized using a funnel plot, which appeared symmetric, suggesting no significant publication bias. Additionally, subgroup analyses were performed to explore potential sources of heterogeneity based on study characteristics, including the sample size, training intensity, and study quality. This process helped to determine whether the overall findings were influenced by any particular subset of studies with higher risk of bias.

## Results

### Study Selection

A total of 1,421 articles were retrieved from four databases: PubMed, Embase, Web of Science, and Scopus. After removing duplicates using EndNote software, 673 articles remained. Following further screening of titles and abstracts, 57 full-text publications were retained for detailed review. Of these studies, 12 lacked controlled outcomes, 15 did not address running economy (RE) or energy expenditure, 11 focused on intervention-related injuries, 6 involved mixed training interventions, and 4 had training duration of less than four weeks. These articles were excluded, leaving 9 studies that were included in the systematic review.

### Study Characteristics

Tables 1 and 2 summarize the studies and outline the key characteristics of the participants involved. The nine studies that met the inclusion criteria are detailed, including their participants’ profiles and running economy data. A total of 168 participants (110 males and 48 females) were included in the analysis, with age ranging from 21.9 to 46.9 years. Maximal oxygen uptake data were recorded, with values ranging from 34.0 to 71.4 ml•kg^-1^•min^-1^. Based on the weighted mean values from the studies reporting participants’ characteristics for each group, minimal differences at baseline were observed for age (38.2 vs. 39.3 years), body mass (81.6 vs. 82.2 kg), body height (1.82 vs. 1.81 m), and VO_2max_ (59.6 vs. 59.3 mL•kg^-1^•min^-1^) between the control and HIIT groups. Seven studies included moderately trained or recreational runners, one study featured well-trained participants, and another examined highly trained runners. Participants were involved in a variety of activities, including triathlons and endurance or recreational distance running.

**Table 1:** Characteristics of training intervention of considered studies.

Study G		Subjects’ characteristics					VO_2max_ (ml•kg^-1^•min^-1^) Training intervention						
		n(M/F)	Age	H	W BMI		Pre	post	PL D Fq	TW	exercise	Load	rest
Reuter et al. (2024)	HIIT	15 (7M; 8F)	45.9 ± 9	171.2 ± 5.3	75 ± 12	25.4 ± 3.5	34.0 ± 5.2	37.3 ± 4.9	MT 26 3	84 min	4 × 4 min	95% HR_max_	3 min
	MICT	16 (6M; 10F)	46.9 ± 7.8	170.6 ± 9.9	75 ± 16	25.5 ± 3.3	34.9 ± 3.6	35.3 ± 4	MT 26 3	150 min	50 min × 1 session	55%HR_reserve_	
Rago et al. (2022)	HIIT	11 (M)	22.0 ± 1.8	184.9 ± 5.4	76.9 ± 5.4	22.49 ± 1.58	60.82 ± 2.66	62.56 ± 3.42	WT 10 2	360 s	6–12 × 30-s sprints	~95%VO_2_	3 min
	MICT	11 (M)	21.9 ± 2.3	182.4 ± 7.9	73.0 ± 6.1	21.94 ± 1.83	60.56 ± 3.10	61.1 ± 3.81	WT 102	20–40 min	2 × 1 week of low-intensity running	70% VO_2max_	
Festa et al. (2020)	HIIT	19 (15M; 4F)	43.2 ± 8.4	175.2 ± 5.9	72.0 ± 7.7	23.46 ± 0.02	52.9 ± 8.1	53.6 ± 4.8	MT 8 4	3.74 ± 0.38 h	2 high Intensities running		
	MICT	19 (16M; 3F)	39.4 ± 8.5	172.5 ± 4.3	70.9 ± 10.1	23.83 ± 0.14	53.4 ± 8.3	53.2 ± 1.9	MT 8 4	3.1 ± 0.25 h	moderate intensity		
Jarstad and Mamen (2019)	HIIT	7 (4M; 3F)	28 ± 5	NR	78.4 ± 11	23.9 ± 2.3	50.6 ± 5.4	53.5 ± 6.9	MT 10 3	60 min	20-min strenuous exhausting run	83% VO_2max_	NR
	MICT	7 (4M; 4F)	28 ± 5	NR	76.3 ± 10.4	24.0 ± 2.7	50.9 ± 7.1	53.3 ± 7.2	MT 10 3	120 min	40-min run at ∼80% HR_max_	72% VO_2max_	NR
Silva et al. (2017)	HIIT	8 (M)	35 ± 6	172.5 ± 4.1	70.5 ± 4.6	NR	54.5 ± 8.1	57.1 ± 6.4	MT 4 2	265 ± 67 s	high-intensity interval training twice weekly		NR
	MICT	8 (M)	32 ± 9	172.8 ± 9	70.2 ± 11.3		56.6 ± 7.3	56.9 ± 7.6	MT 4 2		regular endurance training		
González-Mohíno et al. (2016)	HIIT	6	NR				57.71 ± 8.92	56.2 ± 6.685	MT 6 3	29 min	Interval training 95–110% of MAS	95–110% of MAS	1min
	MICT	5					55.48 ± 8.45	51.66 ± 6.68	MT 6 3	60 min	Interval training 70–75% of MAS	70–75% of MAS	
Gojanovic et al. (2015)	HIIT	6 (M)	31.7 ± 5.2	182 ± 10	77.3 ± 5.2	23.4 ± 2	54.1 ± 8.3	55.1 ± 7.6	MT 4 2	534 s	4–5 intervals run at 100% of velocity to exhaustion	vVO_2max_	267 ± 68 s
	MICT	5 (M)	38.4 ± 9.7	178 ± 4	75.9 ± 3.6	24.1 ± 1.6	57.6 ± 2.5	59.6 ± 2	MT 4 2	368 s			
Enoksen et al. (2011)	HIIT	9 (M)	NR				70.2 ± 2.7	71.4 ± 2.4	HT 10 6	NR	intensive workouts per week at 82–92% of HR_max_	82% HR_max_	NR
	MICT	10 (M)					70.4 ± 3.8	69.2 ± 3.6	HT 10 6	NR	performed at 65–82% of HR_max_.	65% HR_max_	
Litleskare et al. (2020)	HIIT	13 (5M; 8F)	25 ± 1	175 ± 3	71.2 ± 4.1	24.0 ± 0.8	50.5 ± 1.6	53.3 ± 1.5	MT 8 3	150 s	5–10 per session 30-s sprints at near maximal effort	85% HR_peak_	3 min
	MICT	12 (4M; 8F)	25 ± 1	173 ± 2	72.6 ± 3.8	23.6 ± 0.9	47.9 ± 1.5	49.7 ± 1.5	MT 8 3	150 min	70%HR_peak_ training sessions 30 min up to 60 min	70% HR_peak_	

HIIT: high-intensity interval training, MICT: moderate-intensity continuous training, G: group, n: sample size, F: female, M: male, Age (years), H: body height (cm), W: body weight (kg), BMI: body mass index, pre untrained, post trained, VO_2_: maximal oxygen uptake, PL: performance level, D: duration (weeks), Fq: frequency (session/week), TW: Weekly training hours, HT: highly trained, MT: moderately trained, WT: well trained, NR: not reported, MAS: Velocity at VO_2_, V: Velocity, HR_max_: maximum heart rate , HR_peak_: peak heart rate, HR_reserve_: maximum heart rate reserve, s: seconds, min: minute

**Table 2:** Running economy of studies included in the meta-analysis.

Study		G	N	running economy								
				measurement	speed reported	Relative to AT	Relative to VO_2max_ (%)	mean pre (SD)	mean post (SD)	outcomes	
Reuter et al. (2024)	HIIT		15(7M; 8F)	ml•kg^-1^•min^-1^	7	<AT	70% VO_2max_	157 ± 75	152 ± 73	①②③	
	MICT		16(6M; 10F)	ml•kg^-1^•min^-1^	7	<AT	70% VO_2max_	161 ± 50	150 ± 47	①②③	
Rago et al. (2022)	HIIT		11(M)	ml•kg^-1^•min^-1^	10	<AT		224.00 ± 12.84	218.16 ± 11.82	①②	
				ml•kg^-1^•min^-1^	12	≤AT		219.55 ± 13.97	214.74 ± 11.34		
				ml•kg^-1^•min^-1^	14	≤AT		215.17 ± 12.29	216.47 ± 9.70		
	MICT		11(M)	ml•kg^-1^•min^-1^	10	<AT		211.31 ± 18.79	215.29 ± 18.24	①②	
				ml•kg^-1^•min^-1^	12	≤AT		202.84 ± 18.83	206.46 ± 18.75		
				ml•kg^-1^•min^-1^	14	≤AT		207.47 ± 20.58	210.94 ± 20.71		
Festa et al. (2020)	HIIT		19(15M; 4F)	ml•kg^-1^•min^-1^	8.9 ± 0.2	<AT		226.3 ± 35.2	214.3 ± 33.0	①②	
	MICT		19(16M; 3F)	ml•kg^-1^•min^-1^	8.9 ± 0.2	<AT		231.8 ± 9.1	211.6 ± 6.3	①②	
Jarstad and Mamen (2019)	HIIT		7(4M; 3F)	ml•kg^-0.75^•m^-1^	8.9 ± 1.2	<AT	70% VO_2max_	0.721 ± 0.064	0.694 ± 0.064	①②③	
	MICT		7(4M; 4F)	ml•kg^-0.75^•m^-1^	9.6 ± 0.8	<AT	70% VO_2max_	0.664 ± 0.054	0.685 ± 0.058	①②③	
Silva et al. (2017)	HIIT		8(M)	ml•kg^-1^•min^-1^	12	≤AT		43.1 ± 3.5	40.7 ± 4.3	①②	
	MICT		8(M)	ml•kg^-1^•min^-1^	12	≤AT		40.9 ± 4.7	41.2 ± 4.4	①②	
González-Mohíno et al. (2016)	HIIT		6	ml•kg^-1^•min^-1^	10.0 ± 0.88	<AT	60% VO_2max_	37.55 ± 4.92	38.55 ± 4.23	①②	
				ml•kg^-1^•min^-1^	14.53 ± 1.18	≤AT	80% VO_2max_	48.3 ± 5.41	46.81 ± 6.44		
				ml•kg^-1^•min^-1^	16.34 ± 1.32	>AT	90% VO_2max_	52.6 ± 6.96	51.8 ± 5.19		
	MICT		5	ml•kg^-1^•min^-1^	9.84 ± 1.09	<AT	60% VO_2max_	38.46 ± 2.46	32.66 ± 4.54	①②	
				ml•kg^-1^•min^-1^	13.12 ± 1.45	≤AT	80% VO_2max_	45.62 ± 3.77	43.22 ± 3.88		
				ml•kg^-1^•min^-1^	14.76 ± 1.63	>AT	90% VO_2max_	50.86 ± 5.68	46.86 ± 5.77		
Gojanovic et al. (2015)	HIIT		6(M)	ml•kg^-1^•min^-1^	14	≤AT		47.1 ± 4	45.9 ± 4.6	①②	
	MICT		5(M)	ml•kg^-1^•min^-1^	14	≤AT		48.4 ± 4.9	47.3 ± 3.7	①②	
Enoksen et al. (2011)	HIIT		9	ml•kg^-1^•min^-1^	13	≤AT		51.1 ± 3.8	48.7 ± 3.0	①②③	
				ml•kg^-1^•min^-1^	14.5	≤AT		56.9 ± 3.3	55.1 ± 3.2		
				ml•kg^-1^•min^-1^	16	>AT		62.0 ± 3.0	60.4 ± 2.5		
	MICT		10	ml•kg^-1^•min^-1^	13	≤AT		49.6 ± 2.3	47.5 ± 1.7	①②③	
				ml•kg^-1^•min^-1^	14.5	≤AT		55.3 ± 2.7	52.8 ± 2.7		
				ml•kg^-1^•min^-1^	16	>AT		61.4 ± 2.2	59.0 ± 2.6		
Litleskare et al. (2020)	HIIT		13(5M; 8F)	ml•kg^-1^•min^-1^	6.4 ± 0.2	<AT	50% VO_2max_	201 ± 10	199 ± 10	①②③	
				ml•kg^-1^•min^-1^	10.4 ± 0.3	≤AT	80% VO_2max_	237 ± 4	231 ± 4		
	MICT		12(4M; 8F)	ml•kg^-1^•min^-1^	6.2 ± 0.2	<AT	50% VO_2max_	213 ± 16	186 ± 10	①②③	
				ml•kg^-1^•min^-1^	10.1 ± 0.3	≤AT	80% VO_2max_	232 ± 6	223 ± 6		

HIIT: high-intensity interval training, MICT: moderate-intensity continuous training, G: group, N: sample size, F: female, M: male, AT: anaerobic threshold, ① Running Economy, ② Maximal Oxygen Uptake, ③ Blood Lactate

Table 2 provides an overview of the running economy characteristics associated with the outcomes of concurrent HIIT and MICT interventions. Running economy was evaluated by measuring the oxygen cost associated with each condition at a specific running speed. Statistically significant improvements in RE were observed in at least one of the nine studies. Five studies assessed RE at a single speed, while four others evaluated RE at multiple speeds. Additionally, maximal oxygen uptake and blood lactate concentration were included as secondary measures to assess other factors influencing RE.

### Assessment of Quality

Figure 2 shows a statistical chart illustrating the proportion of projects evaluated using different methods. Among the nine articles considered, the blind method posed the highest risk, as the experimental intervention necessitated participants to sign informed consent, leading to significant bias (Figure 2A). Three studies exhibited low risk of bias and high quality, while the remaining six studies had moderate risk of bias.

**Figure 1:**
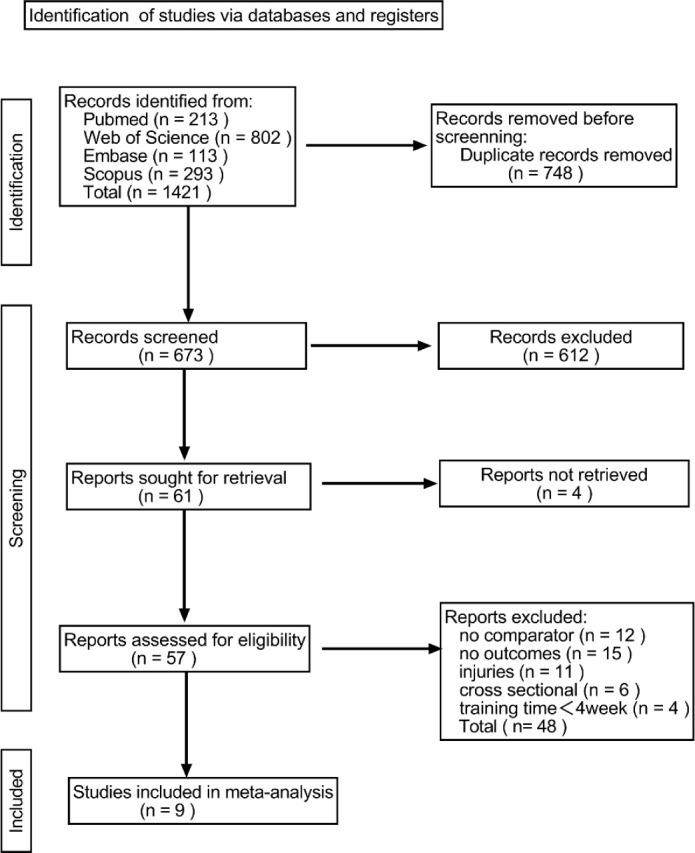
Flow diagram of the search strategy.

**Figure 2:**
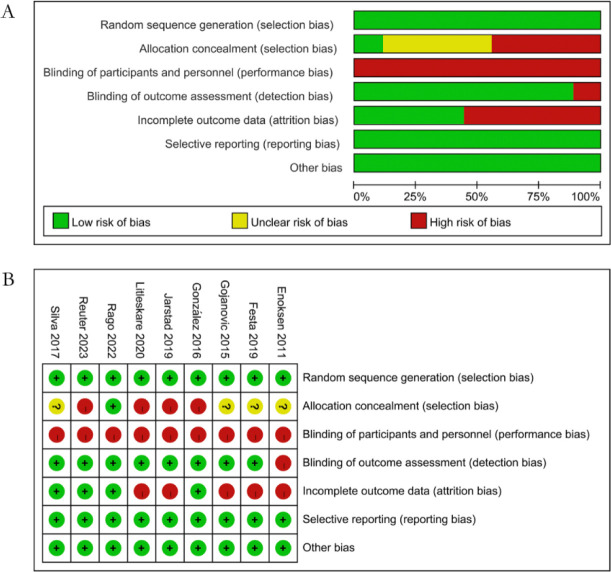
Assessment of quality. Note: In Figure 2(B), the “+” symbol represents low risk of bias, whereas the “–” symbol indicates high risk of bias

### Primary Outcomes

#### Running Economy

This systematic review included nine randomized controlled trials (RCTs) that assessed running economy (RE) at velocities ranging from 7.00 to 16.00 km/h. Due to variability in gender, age, units of measurement, and test intensity, a random effects model was applied. The lactate threshold refers to the point at which blood lactate levels rise nonlinearly. The first lactate threshold (LT1) occurs at approximately 2 mmol•L^-1^, while the second lactate threshold (LT2) is observed at approximately 4 mmol•L^-1^ (Seiler and Kjerland, 2006). The ventilation threshold exhibits a similar pattern, with the first ventilation threshold (VT1) corresponding to the LT1 (approximately 75% VO_2max_) and the second ventilation threshold (VT2) aligning with the LT2 (approximately 85% VO_2max_) (Keir et al., 2022). Based on the research conducted by Casado et al. (2022), this study categorized exercise intensity into three commonly utilized training zones for subgroup analysis: Zone 1 (Z1) corresponded to speeds below the first ventilatory or lactate threshold (approximately 70% VO_2max_), Zone 2 (Z2) included speeds between the first and second thresholds (approximately 75%–85% VO_2max_), and Zone 3 (Z3) encompassed speeds exceeding the second threshold (above 85% VO_2max_) (Casado et al., 2022; Stöggl and Sperlich, 2015). Most of the included subjects in this study had oxygen uptake levels between 50 and 60, and only in two studies participants had oxygen uptake levels around 35 and 70, respectively, which may have impacted the results. Therefore, we conducted sensitivity analysis on these two studies and found that their results did not significantly affect the overall results of the study. In Z1, the heterogeneity among studies was low (I^2^ = 7%, *p* = 0.38). The standardized mean difference (SMD) was 0.40, with a 95% confidence interval of [0.04, 0.76], indicating statistical significance (Z = 2.16, *p* < 0.05). These results suggest that HIIT was more effective than MICT at improving RE at speeds within Z1. In Z2, there was minimal heterogeneity among the studies (I^2^ = 34%, *p* = 0.19). The standardized mean difference (SMD) was 0.68, with a 95% confidence interval of [0.13, 1.24], showing statistical significance (Z = 2.40, *p* < 0.05). The results indicated that subjects following HIIT exhibited better running economy and consumed less oxygen compared to those following MICT at speeds within Z2. In Z3, where speeds exceeded the lactate threshold, there was no intra-group heterogeneity (I^2^ = 40%, *p* > 0.1). The standardized mean difference (SMD) was 0.14, with a 95% confidence interval of [−0.60, 0.88]. There was no statistical significance in training results within Z3 (Z = 0.37, *p* > 0.05), which indicates that there was no significant difference in subjects’ running economy between HIIT and MICT at speeds exceeding the lactate threshold.

The heterogeneity between groups was low (I^2^ = 24%, *p* > 0.1). The standardized mean difference (SMD) between groups was 0.44, with a 95% confidence interval of [0.15, 0.72], indicating statistical significance (Z = 3.01, *p* < 0.05). This indicates that both HIIT and MICT had a greater effect on RE, with the control group showing a larger total effect size than the experimental group. This also suggests that the control group consumed more oxygen under similar conditions, while the experimental group consumed less oxygen. Thus, it can be stated that HIIT was more effective in improving RE.

#### Maximal Oxygen Uptake

Changes in maximal oxygen uptake (VO_2max_) were compared between HIIT and MICT, before and after the experiment. The heterogeneity among the groups was low (I^2^ = 38%, *p* = 0.12), which justified the use of a fixed-effects model. In general, when the I^2^ value is below 50% (or the commonly used threshold is 25%–50%), the heterogeneity is small, the results tend to be consistent, and the use of fixed-effect models is a suitable choice. The mean difference (MD) was 2.48, with a 95% confidence interval of [1.61, 3.34], indicating statistical significance (Z = 5.60, *p* < 0.05). The findings revealed significant differences in VO_2max_ between the two training regimens, with MICT showing a greater improvement compared to HIIT.

#### Blood Lactate Variables

The blood lactate levels were measured at the velocity associated with VO_2max_ in three studies, while fixed blood lactate levels were assessed in one study. The effects of HIIT and MICT on blood lactate levels were evaluated before and after the intervention. There was no heterogeneity among the groups (I^2^ = 0%, *p* > 0.1), and a fixed-effect model was employed for the analysis. The research summary indicated a mean difference (MD) of 0.15, with a 95% confidence interval of [−0.28, −0.02], which was statistically significant (Z = 2.20, *p* < 0.05). The results indicated that lactic acid production in HIIT was lower than that in MICT at the same intensity following training. Additionally, lactic acid tolerance was improved.

### Analysis of Publication Bias

A funnel plot was created to assess running economy as the primary outcome measure. The funnel plot was visually inspected, and it appeared symmetric, suggesting that the included studies were fairly distributed around the pooled effect size. This symmetry typically indicates the absence of significant publication bias, as smaller studies are equally likely to show both positive and negative results. The Egger’s test for asymmetry yielded no significant results (*p* > 0.05), and further statistical tests indicated that the distribution of study effects was likely unbiased. However, it is important to note that while no significant publication bias was detected, small studies with null or negative results may still be underreported, potentially leading to some unobserved bias. Overall, the evidence suggests that publication bias did not significantly affect the findings of this meta-analysis, although caution should always be taken when interpreting results from small studies with non-significant outcomes.

## Discussion

The objective of this systematic review was to critically evaluate the existing literature to determine the effects of HIIT and MICT on running economy (RE). The initial findings revealed that HIIT significantly reduced the oxygen cost of running compared to MICT in endurance runners. Furthermore, the effects of different training interventions on maximal oxygen uptake indicated that MICT was more effective in improving VO_2max_. Finally, training had a significant impact on blood lactate concentration. HIIT was particularly effective as it enabled the body to adapt to lactate more efficiently than most of other forms of exercise. Individuals who engaged in HIIT produced less lactate at similar exercise intensities and demonstrated higher tolerance to lactate.

The objective of this systematic review was to critically evaluate the existing literature to determine the effects of HIIT and MICT on running economy (RE). The initial findings revealed that HIIT significantly reduced the oxygen cost of running compared to MICT in endurance runners. Furthermore, the effects of different training interventions on maximal oxygen uptake indicated that MICT was more effective in improving VO_2max_. Finally, training had a significant impact on blood lactate concentration. HIIT was particularly effective as it enabled the body to adapt to lactate more efficiently than most of other forms of exercise. Individuals who engaged in HIIT produced less lactate at similar exercise intensities and demonstrated higher tolerance to lactate.

**Figure 3:**
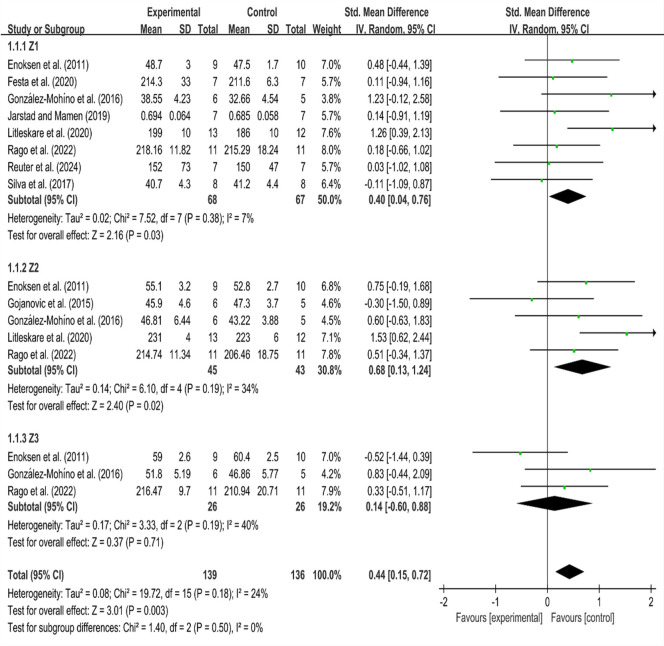
Forest plot for running economy.

Our initial findings showed that, among endurance runners, HIIT led to significantly reduced oxygen cost of running compared to MICT (Wang et al., 2012). Previous studies have indicated that different training interventions can have distinct effects on running economy (Barnes and Kilding, 2015; Faelli et al., 2021; Franch et al., 1998). Compared to MICT interventions, HIIT intervals of 1 min or less did not demonstrate a significant difference in oxygen costs. However, intervals longer than one minute led to a significant improvement in maximal oxygen uptake (Hov et al., 2023). Increasing the work interval from 30 to 60 s led to an increase in maximal oxygen uptake, despite shorter total training duration (Wen et al., 2019). Doubling the interval duration (2 min versus 1 min) resulted in a substantial increase in anaerobic glycolytic energy release, along with an even greater enhancement in oxygen availability (Vuorimaa et al., 2000). HIIT primarily acts on fast-twitch muscle fibers through the arrangement of interval training duration and recovery time (interval training with duration of ≥ 2 min being particularly effective for aerobic stimulation); MICT primarily targets slow muscle fibers, through continuous training, it increases the number of mitochondria and the expression of related proteases in slow muscles (Skelly et al., 2021). It not only enhances the functions of the autonomic nervous system and parasympathetic nervous systems (Clemente-Suárez and Arroyo-Toledo, 2018), but also induces lactic acid accumulation, activates antioxidant enzyme systems, and improves glycolytic capacity through rapid muscle contractions and exertion (Peake et al., 2014). Additionally, HIIT enhances muscle and mitochondrial size (Estes et al., 2017; Ruegsegger et al., 2023), increases hemoglobin levels, and improves blood flow-mediated vasodilation (Mandić et al., 2022; Sawyer et al., 2016). These adaptations contribute to the overall efficiency of both aerobic and anaerobic energy systems (Bogdanis et al., 2013; Buchheit and Laursen, 2013). MICT can increase muscle capillary density, improve myocardial perfusion, enhance blood flow-mediated dilation, and facilitate oxygen delivery (Cocks et al., 2016; Daussin et al., 2008). These effects are beneficial for energy supply during exercise and contribute to the body’s long-term resistance to fatigue (Wang et al., 2021). The spring-mass model is an important factor associated with RE, where the body’s bounce on the ground is counteracted by the spring-like behavior of the supporting leg. Mechanical energy is stored in the muscles, tendons, and ligaments acting across the joints (Saunders et al., 2004). HIIT can have greater impact on muscle elasticity than MICT, the faster the speed, and the higher the stiffness in the lower limb muscles. The greater the stiffness, the more beneficial it is for runners to consume less oxygen (Liu et al., 2022). Compared with MICT, HIIT brings greater physical improvements and adaptive changes over a short period of time, thereby enhancing RE of runners (Skovgaard et al., 2018a). According to our meta-analysis, HIIT has more beneficial effects on RE at intensities near Z2 compared to those near Z3. When the velocity exceeds Z2, the effect size becomes non-significant (Figure 3). Training at an intensity close to Z2 may elicit a combination of molecular signals generated by both high-volume and high-intensity training. This combination of signals provides greater stimulation and has a more significant impact on the body’s adaptation, which is more conducive to improving RE (Skovgaard et al., 2018b). Training in Z3 primarily stimulates the body anaerobically, and although this intensity can also stimulate the body’s aerobic metabolism to a certain extent, the effect is inferior to the former. This finding is consistent with research indicating that when lactate concentration is greater than 4 mmol/L, the enhancement in running economy diminishes (Zinner et al., 2018). Furthermore, the impact on running economy continues to decline as exercise intensity increases (Munoz et al., 2015). Among the different runners, we found that the improvement in running economy within the Z3 was reduced for well-trained and recreational runners, with the effect being particularly diminished for recreational runners. Repeated training in Z3 may result in muscle damage, fatigue, and decreased parasympathetic nerve activity (Kingsley et al., 2005; Nuuttila et al., 2022). For runners, training intensity should be kept close to Z2 to optimize running economy, especially for low- and medium-level athletes. Overall, our findings indicate that HIIT at or below the lactate threshold during the Z2 phase is essential for enhancing running economy.

**Figure 4:**
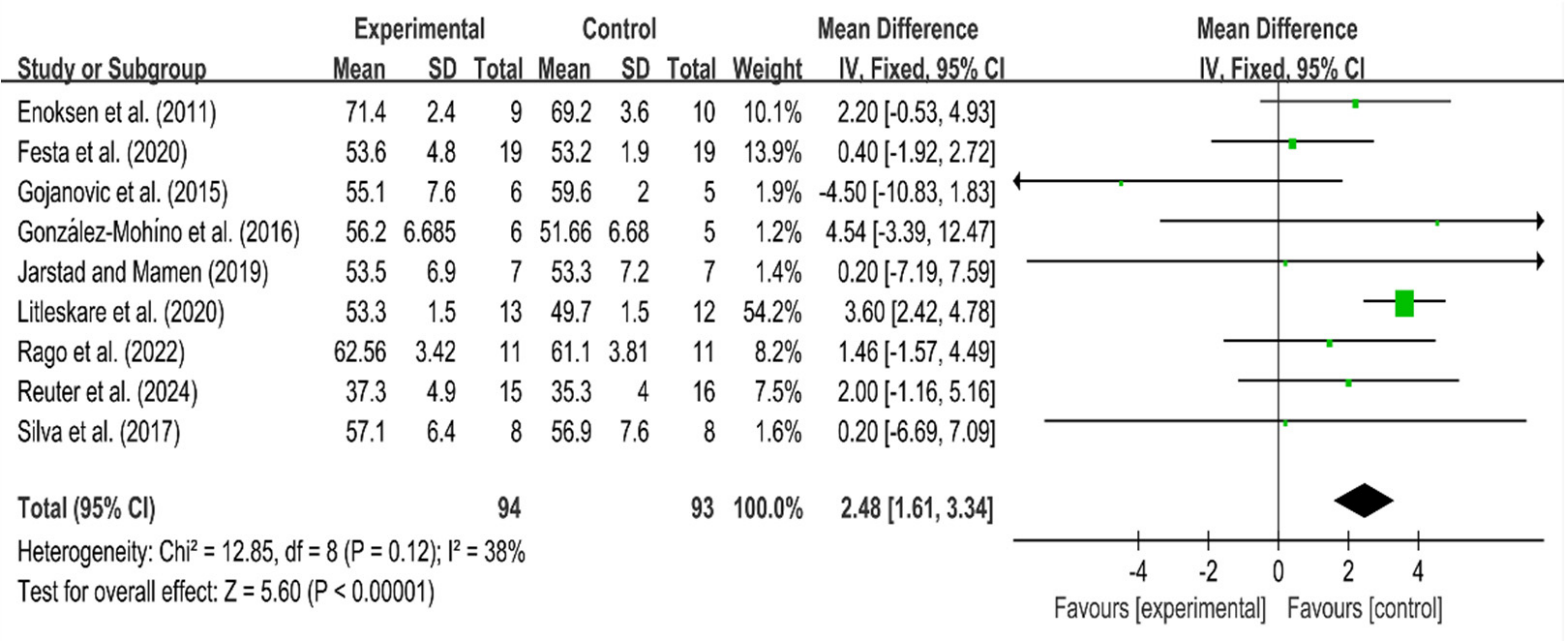
Forest plot for VO_2max_.

**Figure 5:**
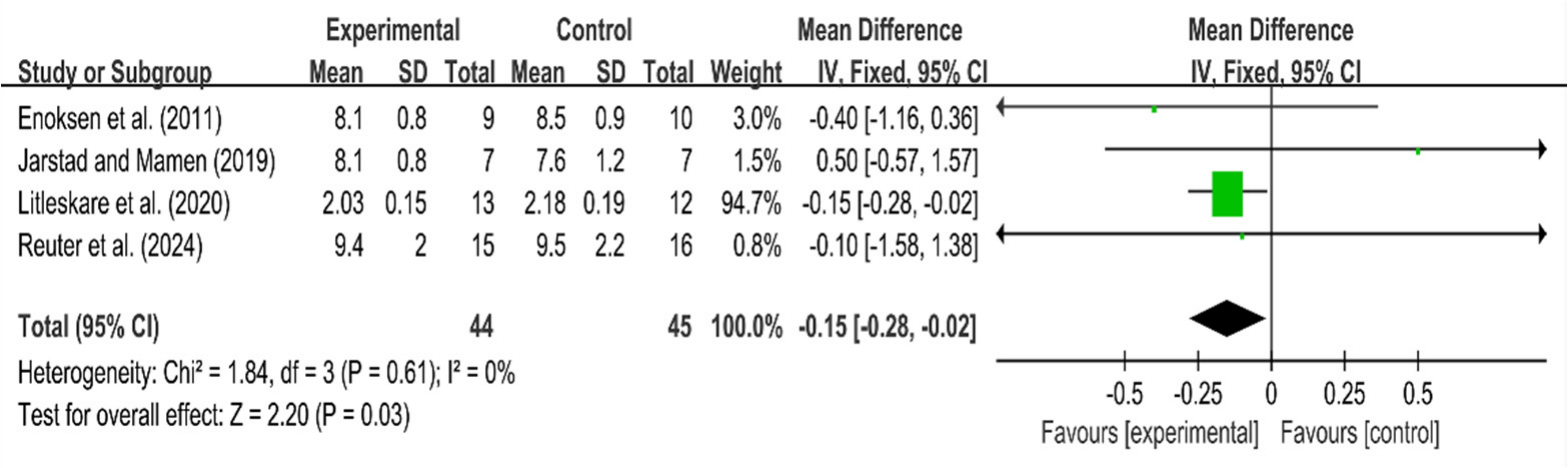
Forest plot for blood lactate concentration.

**Figure 6:**
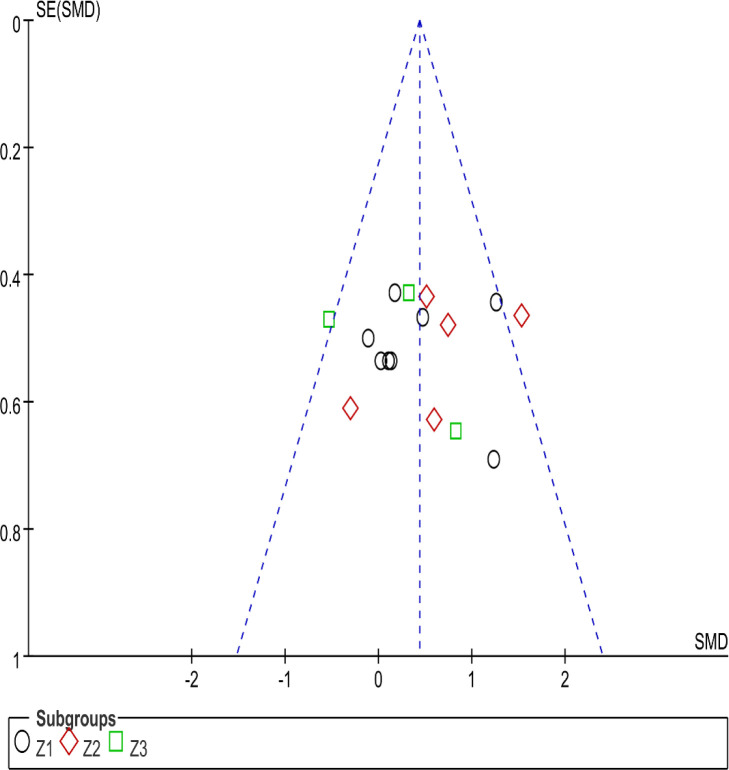
The risk of bias funnel plot.

It is widely acknowledged that maximal oxygen uptake is one of the most critical factors for successful distance running (Parmar et al., 2021). Current studies have shown that both HIIT and MICT interventions can enhance maximal oxygen uptake (Mølmen et al., 2025). Our meta-analysis found that MICT had a greater effect on peak oxygen uptake than HIIT, which is consistent with a recent review of related studies (Guo et al., 2022; Liang et al., 2024). However, other scholars have expressed an opposing viewpoint. Some researchers pointed out that HIIT was particularly effective in improving VO_2max_ during the initial stages of training for individuals with lower fitness levels (Mølmen et al., 2025). However, improvements in ventilatory thresholds above the second ventilatory improvements were only observed in well-trained endurance runners (Seiler et al., 2007; Tanji and Nabekura, 2019). HIIT can cause muscle damage and pain, increase cellular damage, lipid peroxidation, inflammatory response, and adversely affect muscle cell structure and the function of contractile proteins (Kingsley et al., 2005; Leite et al., 2023). HIIT for recreational runners may increase their risk of muscle injuries (Leite et al., 2023; Nuuttila et al., 2022). Additionally, it can lead to decreased adherence to training, particularly in unsupervised settings (Ekkekakis and Biddle, 2023; Santos et al., 2023). In contrast, MICT is relatively effective in mitigating these risks and enhancing runners’ compliance with their training regimens (Ekkekakis and Biddle, 2023; Santos et al., 2023). Our research indicates that MICT is a more effective way to develop aerobic endurance than HIIT. For most runners, particularly beginners and those who are untrained, utilizing MICT to enhance aerobic capacity still has its significant advantages.

Blood lactate concentration is an important indicator of an individual’s exercise intensity. Distance running performance can be partially predicted by blood lactate concentration at a specific running speed (Farrell et al., 1979; Fay et al., 1989; Yoshida et al., 1993). This research included four studies on blood lactate concentration, which demonstrated that HIIT was more effective in adapting blood lactate concentration than MICT. This may be attributed to an increased muscle oxidative capacity and associated changes in motor unit recruitment patterns, as well as muscle adaptations, enhanced glycolytic capacity, increased oxidative activity, and alterations in lactate transport (Denadai et al., 2006; Joyner and Coyle, 2008). These benefits include enhanced recovery of creatine phosphate and increased mitochondrial biogenesis, improved skeletal muscle buffering capacity, enhanced ventilation, and elevated lactate threshold (Driller et al., 2009; Esfarjani and Laursen, 2007; Hoogeveen, 2000; Weston et al., 1997). Additionally, there is an increased capacity to engage larger volumes of muscle mass (Creer et al., 2004) and a greater ability to oxidize fat relative to carbohydrates (Atakan et al., 2022). Compared with MICT, HIIT can delay lactic acid accumulation in the blood and postpone muscle fatigue during high-intensity exercise, resulting in improved physical performance and improved training outcomes.

Both funnel plot and the Egger’s test showed no obvious publication bias for the included studies. These studies exhibited diversity in terms of the study sites, populations, and designs, which reduced the likelihood of publication bias to some extent. Since different study settings and designs produce different effect size estimates, it is difficult to guarantee a consistent positive outcome preference across such diverse studies if publication bias exists. We found some differences in the maximum oxygen uptake of Reuter et al. (2024) and Enoksen et al.’s (2011) studies, which were about 35 and 70, respectively. This difference may bias the results of the studies, thus sensitivity analysis was conducted one by one. It was found that the inclusion and exclusion of these two articles had no significant effect on the results of the study. We weighted each study based on its quality, sample size, and other factors. Despite potential bias in some studies (e.g., differences in oxygen uptake between Reuter et al. (2024) and Enoksen et al.’s (2011) studies), sensitivity analysis showed that including or excluding these studies did not significantly affect the final results. Despite some bias, the weighting method and analysis results remained stable and reliable.

The limitations of this systematic review include methodological and statistical heterogeneity among the included studies, with some exhibiting risk of bias due to methodological weaknesses, such as lack of blinding in intervention studies. Varying measurement methods may have contributed to heterogeneous results, and although subgroup analysis addressed some sources of heterogeneity, individual-level moderators remained. Furthermore, the studies did not account for other influencing factors, such as environmental conditions, sleep, diet, and psychological stress, which can affect running economy. For instance, acute sleep deprivation can impair performance (Craven et al., 2022), leading to an increased heart rate and minute ventilation during exercise (Mougin et al., 1991). Poor sleep can also deteriorate athletic performance, increasing energy cost during exercise (Vitale et al., 2019). Additionally, moderate carbohydrate intake before and after exercise can improve endurance and glycogen reserves (Flockhart and Larsen, 2024; Khanna and Manna, 2005), while proper protein supplementation aids muscle synthesis and repair (Phillips and Van Loon, 2011). Dietary intake of carbohydrates and fats influences fuel utilization and running economy (Maunder et al., 2021), with low carbohydrate (<20%) and high fat (>60%) intake promoting fat oxidation and improved submaximal running economy (Che et al., 2021). Psychological stress can also impact performance by altering hormone levels, such as cortisol and adrenaline (Rimmele et al., 2009; Schommer et al., 2003). Pre-match anxiety can elevate the heart rate and blood pressure, impairing energy utilization and performance (Nibbeling et al., 2012; Webb et al., 2008). Lastly, the review was restricted to English-language studies and to the sport of running, which may limit the generalizability of the findings. Therefore, caution is needed when interpreting the results, and future research should explore running economy in other sports and consider papers published in other languages for broader applicability.

HIIT has gained popularity, but studies comparing its long-term effects with MICT show no significant superiority. While short-term compliance with HIIT is higher (Jung et al., 2015), long-term adherence drops significantly, with dropout rates higher than MICT (Roy et al., 2018). One study found adherence dropping from 82% to 29% at 12 months. During a 12-month follow-up, MICT participants were more likely to complete training (Taylor et al., 2020). HIIT also carries higher risks, with the incidence of acute cardiac events nearly six times higher (Rognmo et al., 2012), and muscle function impairment linked to elevated blood lactate concentration (Thomas et al., 2004). The injury risk associated with different interval duration and intensities during HIIT may lead to musculoskeletal pain, especially at higher running speeds (Leite et al., 2023; Wiewelhove et al., 2016). Therefore, the high intensity, high risk of injury, and low long-term adherence of HIIT make it more challenging for long-term sustainability in mass fitness participants.

## Conclusions

The results of this study suggest that HIIT is a more effective method for improving running economy and delaying lactate accumulation, as well as enhancing lactate tolerance, compared to MICT in long-distance runners. By training close to the lactate threshold (Zone 2), HIIT facilitates greater improvements in running economy and postpones lactate accumulation in the bloodstream. However, improvements in maximal oxygen uptake (VO_2max_) were significantly more pronounced with MICT than with HIIT. Further high-quality studies are needed to investigate these effects in greater depth in the future.
